# AuNP-M2e + sCpG vaccination of juvenile mice generates lifelong protective immunity to influenza A virus infection

**DOI:** 10.1186/s12979-019-0162-y

**Published:** 2019-09-02

**Authors:** Lynn Bimler, Amber Y. Song, Duy T. Le, Ashleigh Murphy Schafer, Silke Paust

**Affiliations:** 1 Center for Human Immunobiology, Department of Pediatrics, Texas Children’s Hospital, Houston, TX 77030 USA; 2 Graduate Program in Immunology, Baylor College of Medicine, Houston, TX 77030 USA; 3 Developing Investigative Scholar’s Program (DISP), Rice University, Houston, TX 77030 USA; 4 Department of Immunology and Microbiology, The Scripps Research Institute, Immunology Building 313/114, 10466 North Torrey Pines Road, La Jolla, California, 92037 USA; 5 Department of Molecular Virology and Microbiology, Baylor College of Medicine, Houston, TX 77030 USA

**Keywords:** Nanoparticle, M2e, Influenza a, Vaccine, Geriatric

## Abstract

**Background:**

Influenza virus infection causes significant morbidity and mortality worldwide. Humans fail to make a universally protective memory response to influenza A because of high mutation rates in the immune-dominant influenza epitopes. We seek the development of a universal influenza A vaccine. The extracellular domain of the M2-ion channel (M2e) is an ideal antigenic target, as it is highly conserved, has a low mutation rate, and is essential for viral entry and replication. Considering the potential of a universal influenza vaccine for lifelong protection, we aimed to examine this potential using a recently published gold nanoparticle M2e vaccine with CpG as an adjuvant (AuNP-M2e + sCpG). Intranasal vaccination induces an M2e-specific memory response, which is protective against lethal infection with H1N1, H3N2, and H5N1 serotypes, in young BALB/c mice. Protection with AuNP-M2e + sCpG has been published up to 8 months after vaccination. However, the highest risk population during most influenza seasons is adults over 65 years old. Additionally, the efficacy of many vaccines decrease after aging and requiring booster vaccinations to remain effective.

**Results:**

To determine if the AuNP-M2e + sCpG vaccine is a viable option as a universal vaccination capable of protection through geriatric age, we tested if the AuNP-M2e + sCpG vaccination loses efficacy after aging mice to geriatric age (over 18 months). Our data shows that mice aged 15 months after vaccination (~ 18–21 months old) retain significant M2e-specific antibody titers in total IgG, IgG1, IgG2a, and IgG2b. These mice are significantly protected from lethal influenza challenge (H1N1, 8.3 PFU). Further, these antibody titers increase upon infection with influenza A and remain elevated for 3 months, suggesting the elderly mice retain effective M2e-specific memory B cells.

**Conclusions:**

Our results demonstrate that protective M2e-specific memory in mice developed at a young age can persist until geriatric age. Additionally, this memory is protective and M2e-specific B cells produced by vaccination with AuNP-M2e + sCpG are maintained and functional. If the results of this study persist in humans, they suggest that a universal influenza A vaccine could be administered early in life and maintain lifelong protection into geriatric age.

## Background

Worldwide up to 650,000 people die from influenza each year, including an average of 42,000 people within the United States, approximately 80% of whom are above the age of 65 [1–3]. High rates of hospitalization and death occur despite seasonal vaccines and available therapies [1, 4]. The influenza vaccine must be updated annually because influenza virus lacks proofreading mechanisms during replication resulting in a high mutation rate. These mutations are especially prevalent in the most immunogenic proteins of influenza, and their accumulation is referred to as antigenic drift. [5, 6] The seasonal influenza vaccine has been utilized in the United States since 1945, but the necessity of reformulating the seasonal vaccine annually is an expensive and time consuming collaborative global effort [3, 7].

Despite extensive screening and development each year, the seasonal vaccine’s efficacy can be limited. Dependent on the degree of mutation after the selection of the vaccine virus sequence each year, efficacy ranges between 10 and 60% [7, 8]. For example, during the 2013–2014 influenza season, the vaccine was 52% effective and vaccinated adults were 52–79% less likely to die as a result of influenza; however, the next year (2014–2015), effectiveness of the vaccine dropped to 19% [8–10].

Influenza A is capable of an additional mechanism of change, antigenic shift. Antigenic shift is the rapid change and development of a new influenza virus and occurs when two different serotypes of influenza A co-infect the same cell and exchange RNA segments [11]. This genetic recombination usually involves a newly human-adapted hemagglutinin (HA) (e.g. from birds or swine) or a highly mutated HA to which the human population is naïve [5, 11]. If antigenic shift occurs after strain selection, there is little to no protection against a potentially pandemic influenza strain [7]. This was the case in 2009, when the A/California/04/2009 pandemic virus dramatically shifted, becoming more antigenically similar to the 1918 “Spanish flu” than to the seasonal H1N1 strains between 1977 and 2008 [12].

Despite the clear need for a vaccine that is universally applicable to seasonal and pandemic strains of influenza A, no universal influenza vaccine has been FDA approved. The AuNP-M2e + sCpG vaccination, first published in 2014, utilizes M2e as a potentially universal target for influenza A, because of the high level of conservation in the M2e peptide sequence between serotypes and isolates and its expression on both the surface of virions and infected cells [12–16]. M2e is the extracellular N-terminal portion of M2 and has been considered an excellent candidate for influenza A vaccination or treatment since its discovery by Lamb, et al. in 1981 [11, 12, 17]. However, there has been limited success in actualizing that potential [18]. Well over 30 M2e vaccines have been developed and published using a variety of adjuvants, including four which have entered clinical trials [12, 19].

AuNP-M2e + sCpG vaccination seems particularly promising because it is easy and inexpensive to produce, has a short manufacturing time, is egg-free, and can be lyophilized so that it is stable long term at room temperature [15], making it not only easy to stockpile but also feasible for large scale production. This vaccine is demonstrated to be very efficacious 21 days post vaccination in protecting against lethal challenge with H1N1 A/PR/8/1934, pH1N1 A/CA/04/2009, H3N2 A/Victoria/3/75, and H5N1 A/Vietnam/1203/2004, indicative of a highly cross reactive memory immune response [15, 20]. The vaccine has also been demonstrated to maintain elevated but gradually decreasing antibody titers and to be protective against H1N1 A/PR/8/1934 lethal challenge up to eight months post vaccination in mice [21]. These publications successfully demonstrate that intranasal vaccination with AuNP-M2e + sCpG in healthy, young 12–14 week old BALB/c mice (vaccinated at 6–8 weeks and infected 42 days post vaccination) and healthy, adult 54–56 week old BALB/c mice (vaccinated at 6–8 weeks and infected 8 months post vaccination) induces an M2e-specific memory response, which is protective against lethal challenge.

However, adults over 65 years old are at the highest risk during most influenza seasons, constituting 71–85% of deaths and 54–70% of hospitalizations related to seasonal influenza, estimated by the CDC [2]. Many studies have identified being over 65 years of age as one of the most significant risk factors for fatality from influenza infection A from current circulating strains of H3N2 and pH1N1 [22, 23]. This seems to be predominantly caused by immunosenescence, or the decreased efficiency of the immune system as a result of aging [24]. In response to influenza this is characterized as decreased antibody-mediated and cellular immunity and decreased responsiveness to vaccines, as decreased thymus function limits the induction of new and memory responses to antigens [24–26]. Adults over 65 typically experience altered clinical presentation of influenza A with reduced fever symptoms but increased respiratory symptoms, including coughing and wheezing [26]. These patients also have increased rates of deadly complications, namely pneumonia to which people over 65 are already at increased risk [26, 27].

Further, many vaccines lose efficacy during aging resulting in a partial loss of protection and potentially requiring booster vaccinations to remain effective [28]. Regular boosters are required for tetanus and diphtheria vaccines, and boosters for pertussis and polio are often recommended [24, 29, 30]. To determine if the AuNP-M2e + sCpG vaccine is a viable option as a universal vaccination and to begin to test it if might require re-administrations during a lifetime, we tested if the AuNP-M2e + sCpG vaccination loses efficacy after aging mice to geriatric age (defined as 18 months or 72 weeks old) [31].

We vaccinated BALB/c mice at 3–6 weeks of age and challenged them 15 months after vaccination with a lethal challenge of H1N1 A/PR/8/1934. At the time of challenge, these mice were approximately 18–24 months old and retain significant M2e-specific antibody titers in total IgG, IgG1, IgG2a, and IgG2b. Further, the antibody titers increase upon infection with influenza A (H1N1 PR8) and remain elevated for at least 3 months, suggesting the elderly mice retain effective M2e-specific memory B cells. These mice are significantly protected from lethal influenza challenge (H1N1, 8.3 PFU). These results suggest that AuNP-M2e-CpG is an excellent candidate as a universal influenza vaccine as it maintains lifelong protection in mice despite aging to geriatric age.

## Results

### Repeated AuNP-M2e + sCpG vaccination of BALB/c mice induces long-term M2e-specific antibody titer

**Fig. 1:**
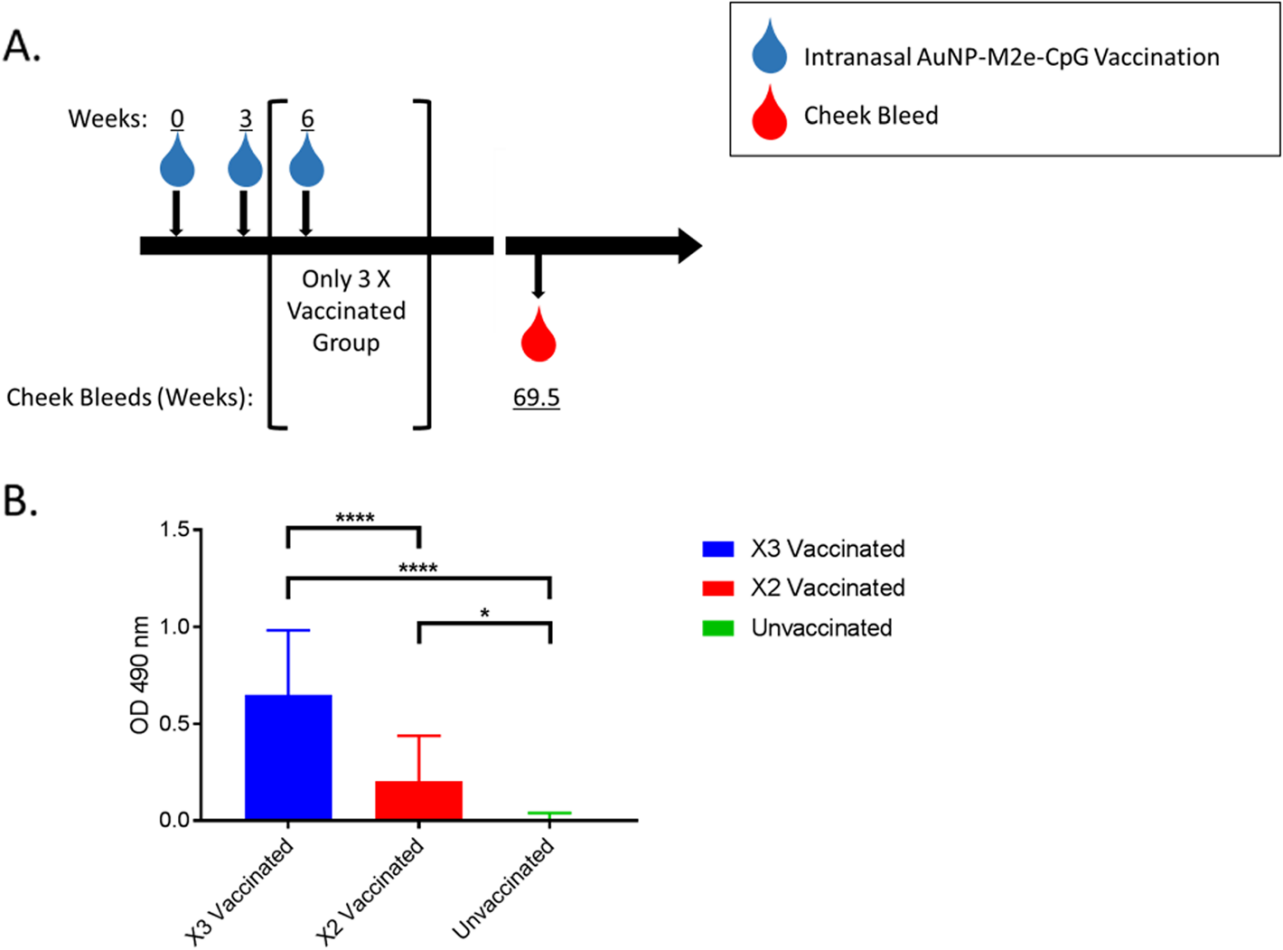
AuNP-M2e + sCpG induces long-term M2e-specific antibody titer. (**a**) A cohort of mice were vaccinated 0, 2, or 3 times with AuNP-M2e + sCpG vaccine and bleed at week 69.5. Diagram shows experimental design. (**b**) Antibody titer determined by ELISA. M2e peptide was used as the coating antigen for ELISAs. Serum from specified mice and time point was added. M2e-specific titer was detected by an IgG subclass specific secondary antibody. Average background from naïve unvaccinated serum was subtracted out. OD 490 nm = Optical Density 490 nm. *n* = 18–19, One-way ANOVA with Tukey’s multiple comparison test. * *p* < 0.05, ** *p* < 0.01, *** *p* < 0.001, and **** *p* < 0.0001

We measured the total M2e-specific IgG in the serum of geriatric aged mice 15 months post-third vaccination with AuNP-M2e + sCpG via M2e peptide ELISA (Fig. 1a). Mice retained M2e-specific IgG in both two and three times vaccinated groups (Fig. 1b). Further, mice vaccinated three times had significantly more M2e-specific total IgG than mice vaccinated twice. This suggests that not only do mice vaccinated at an early age maintain an M2e-specific antibody titer through geriatric age, but that additional boosts at an early age can significantly elevate the M2e-specific titer of geriatric mice after aging 15 months post vaccination.

### Repeated au-NP-M2e + sCpG vaccination of BALB/c mice induces long-term M2e-specific antibody titers of all IgG subclasses

**Fig. 2:**
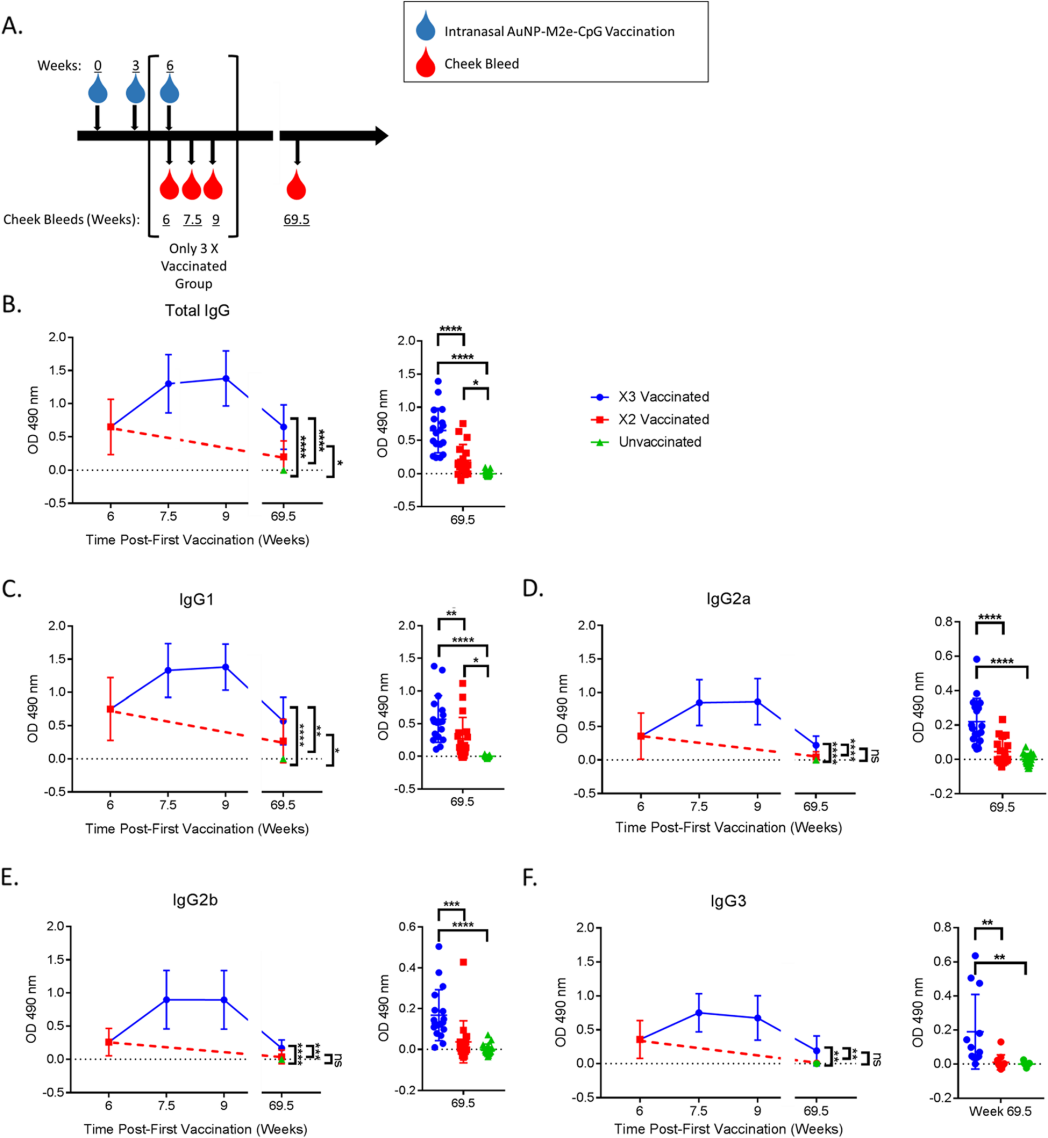
AuNP-M2e + sCpG induces long term titers for all IgG subclasses. (**a**) Diagram containing all time points for this Fig. (**b**-**f**) M2e peptide was used as the coating antigen for ELISAs. Serum from specified mice and time point was added. M2e-specific titer was detected by an IgG subclass specific secondary antibody. Average background from naïve unvaccinated serum was subtracted out. OD 490 nm = Optical Density 490 nm. (**b**-**e**) n = 18–19 and (**f**) *n* = 11–12, One-way ANOVA with Tukey’s multiple comparison test. * p < 0.05, ** *p* < 0.01, *** *p* < 0.001, and **** *p* < 0.0001

We further analyzed the effect of the third AuNP-M2e + sCpG vaccination by monitoring serum titer immediately post vaccination and 15 months post-vaccination (Fig. 2a). Mice vaccinated three times develop strong M2e-specific IgG responses, which are boosted immediately after vaccination and remain elevated through geriatric age (Fig. 2b). This is consistent previous publications with the AuNP-M2e + sCpG vaccination by Tao et al. in 2015, in which the antibody titer boosted immediately after vaccination, increased through Day 21 post-second vaccination, and decreased in the 8 months after vaccination [21]. Here we add to the previously published data by showing that this M2e-specific titer is actually maintained though at least 15 months post vaccination.

The publication by Tao, et al. showed that IgG1 and IgG2a levels are individually elevated post-vaccination and decrease over the next 8 months; here, we show that these antibody titers are maintained through geriatric age in mice vaccinated three times (Fig. 2c and d) [21]. Further the M2e-specific total IgG titer is predominantly IgG1, IgG2a, and IgG2b subclasses (Figs. 2c-e).

Lastly, similar to results in M2e-specific total IgG (Figs. 1b and 2b), M2e-specific IgG1, IgG2a, IgG2b and IgG3 subclass titers are all significantly increased in mice vaccinated with AuNP-M2e + sCpG three times compared to those vaccinated twice or unvaccinated controls. Mice vaccinated two times maintain significantly elevated M2e-specific total IgG and IgG1, but lose significance in M2e-specific IgG2a, IgG2b and IgG3 titers over controls (Figs. 2b-f).

Overall, it is evident that geriatric mice retain significant M2e-specific antibody titers even 15 months post-vaccination with AuNP-M2e + sCpG. The vaccine is capable of maintaining antibody titers representing a variety of IgG subclasses, specifically IgG1, IgG2a, and IgG2b. Further, the number of vaccinations at an early age contributes to the M2e-specific antibody titer of all elevated IgG subclasses after aging.

### AuNP-M2e + sCpG vaccination in juvenile mice induces protection from lethal influenza a H1N1 challenge at geriatric age

**Fig. 3:**
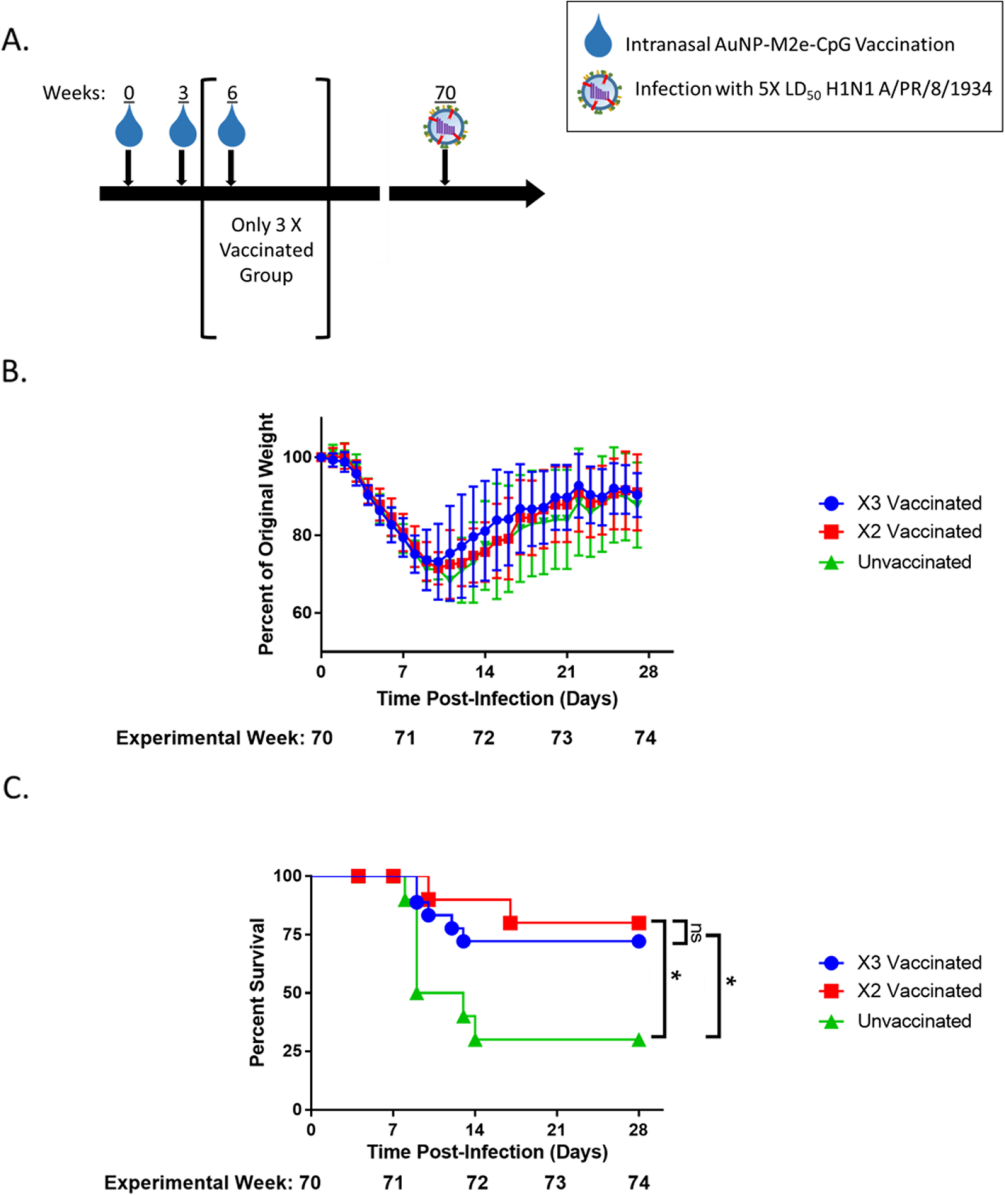
AuNP-M2e + sCpG vaccination protects from lethal H1N1 infection. (**a**) Diagram containing all time points for this Fig. (**b**) Weight loss was monitored daily and percent weight loss was determined utilizing Day 0 Weight. (**c**) Survival was monitored daily. n = 18–19 (9 mice from both two times vaccinated and unvaccinated groups were sacrificed for viral titers during the experiment and were censored within survival data at that time point), log-rank Mantel-Cox Test. * p < 0.05

Knowing that M2e-specific antibody titers are maintained in AuNP-M2e + sCpG vaccinated mice though geriatric age and that these titers are dependent on the number of vaccinations given at an early age, we sought to determine if these elevated M2e-specific antibody titers were indicative of maintained protection from influenza infection. We used A/PR/8/34 (H1N1) which contains one amino acid difference compared to M2e consensus sequence (D21G) and four distinctions compared to the M2e vaccine sequence (S17C, S19C, D21G, and no additional C on the C-terminal). We infected three and two times vaccinated geriatric mice with 8.3 PFU of A/PR/8/34 (H1N1) at experimental week 70, over 15 months after their last AuNP-M2e + sCpG vaccination, and compared their survival and weight loss to infected unvaccinated controls (Fig. 3a). While we did not observe a significant difference in weight loss between groups (Fig. 3b), mice vaccinated two or three times 15 months prior to infection were both significantly protected from mortality compared to unvaccinated controls (Fig. 3c). Therefore, the AuNP-M2e + sCpG vaccine is protective throughout the lifetime of the mouse and retains protection after aging.

It is also worth noting that all mice viral titers in two times vaccinated and unvaccinated mice (*n* = 4) were negative at day 7 post infection (at dilutions ranging from 1:5 to 1X10^− 2^, data not shown). These results are consistent with the literature as BALB/c mice have been established to clear infections with 10^3^ initial viral loads by day 7 [32]. Because most deaths occur after day 7, it is likely that the protective immune response from the vaccine is preventing tissue damage and/or other symptoms and pathologies of influenza infection to which these mice eventually succumb.

### AuNP-M2e + sCpG vaccination in mice induces long lived M2e-specific B cells

**Fig. 4:**
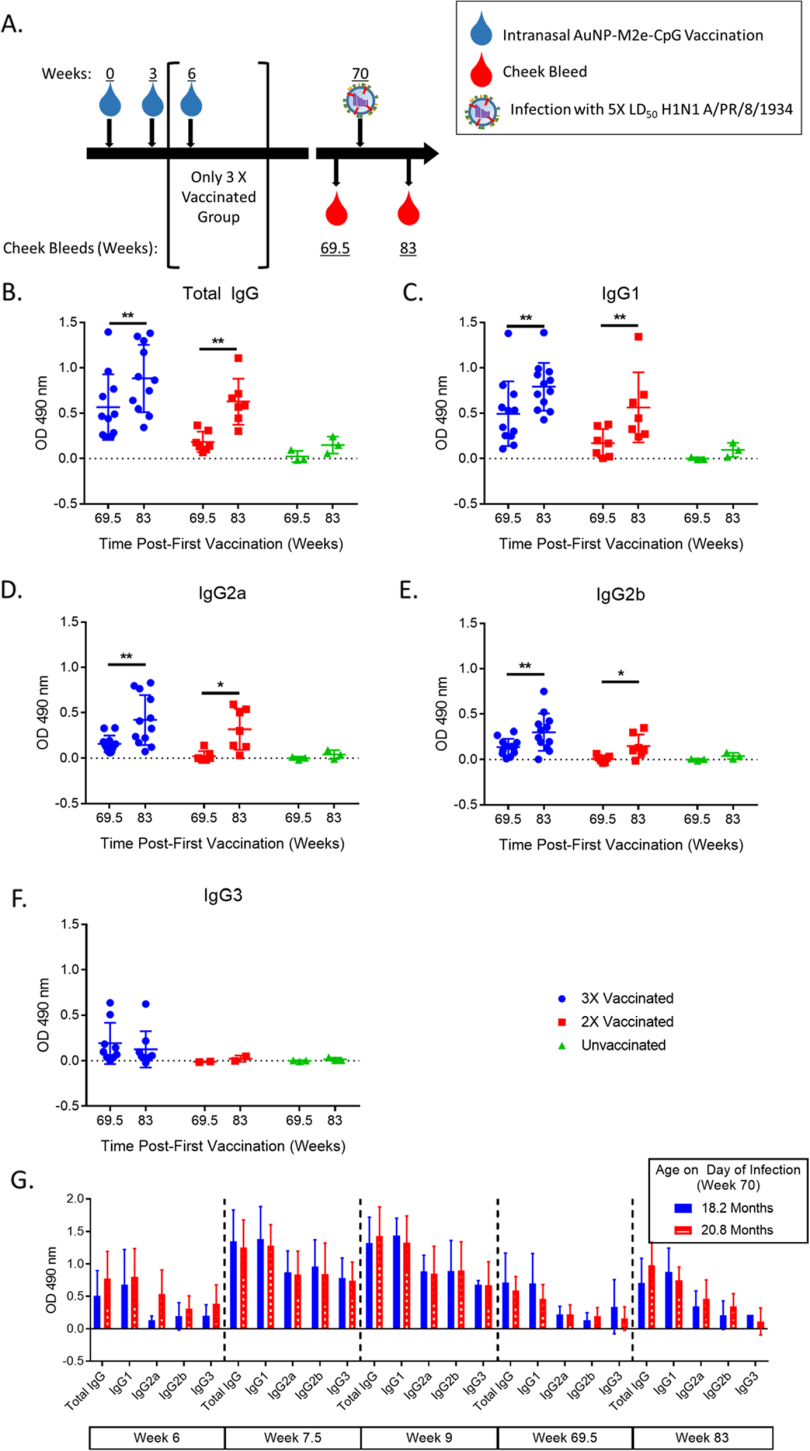
AuNP-M2e + sCpG vaccination induces long lived M2e-specific B cells. (**a**) Diagram containing all time points for this Fig. (**b**-**f**) M2e peptide was used as the coating antigen for ELISAs. Serum from survivor mice from specified group and time point was added and M2e-specific titer was detected by an IgG subclass specific secondary antibody. Average background from naïve unvaccinated serum was subtracted out. OD 490 nm = Optical Density 490 nm. (**b**-**e**) *n* = 3 (Unvaccinated), 7 (2X) and 12 (3X) and (**f**) n = 3 (Unvaccinated), 2 (2X) and 9 (3X). (**b**-**f**) Paired T test. (**g**) Two groups of BALB/c mice (3.4 and 0.8 months at the time of the first vaccination) were vaccinated three times with AuNP-M2e + sCpG on the same days. Antibody titer tested by ELISA as described for B-F. *n* = 8–10 prior to infection for all time points, IgG subclasses, and groups, except 18.2 month old IgG3 (*n* = 2–3) and post-infection n varies between 1 and 8, Two-way ANOVA with Sidak’s multiple comparison test. * *p* < 0.05, ** *p* < 0.01

To examine the M2e-specific response to infection, we compared serum M2e-specific antibody levels from immediately before infection (week 69.5) and 3 months after infection (week 83) in mice that survived the lethal influenza infection (Fig. 4a). The mice vaccinated three times prior to aging had a significant increase in circulating total IgG and all IgG subclasses, except for IgG3, as a result of infection. Mice vaccinated twice had similar results with a significant increase in total IgG, IgG1, IgG2a and IgG2b (Figs. 4b-f). This data shows that geriatric mice retain effective memory B cells 15 months after AuNP-M2e + sCpG vaccination, which are activated and boosted by infection as IgG levels remain elevated 3 months after infection. Of note, unvaccinated survivors appear to develop a slight M2e-specific antibody titer as a result of infection, but the difference in these titers did not achieve significance.

Additionally, the function of M2e-specific memory B cells appears to be dependent on time since vaccination. We initially separately evaluated two sets of mice vaccinated 3 times, one group 2.6 months older than the other. The two groups of mice vaccinated three times with AuNP-M2e + sCpG on the same days, but different ages (3.4 and 0.8 months at the time of the first vaccination). We found no significant difference between the titers of these mice at any time point (Fig. 4g). Therefore, we combined the groups for all other analyses. These data suggest that post-vaccination the changes in titer over the mouse’s lifetime appear to be completely dependent on time since vaccination, rather than age.

### AuNP-M2e + sCpG vaccination induces antibody-mediated protection

**Fig. 5:**
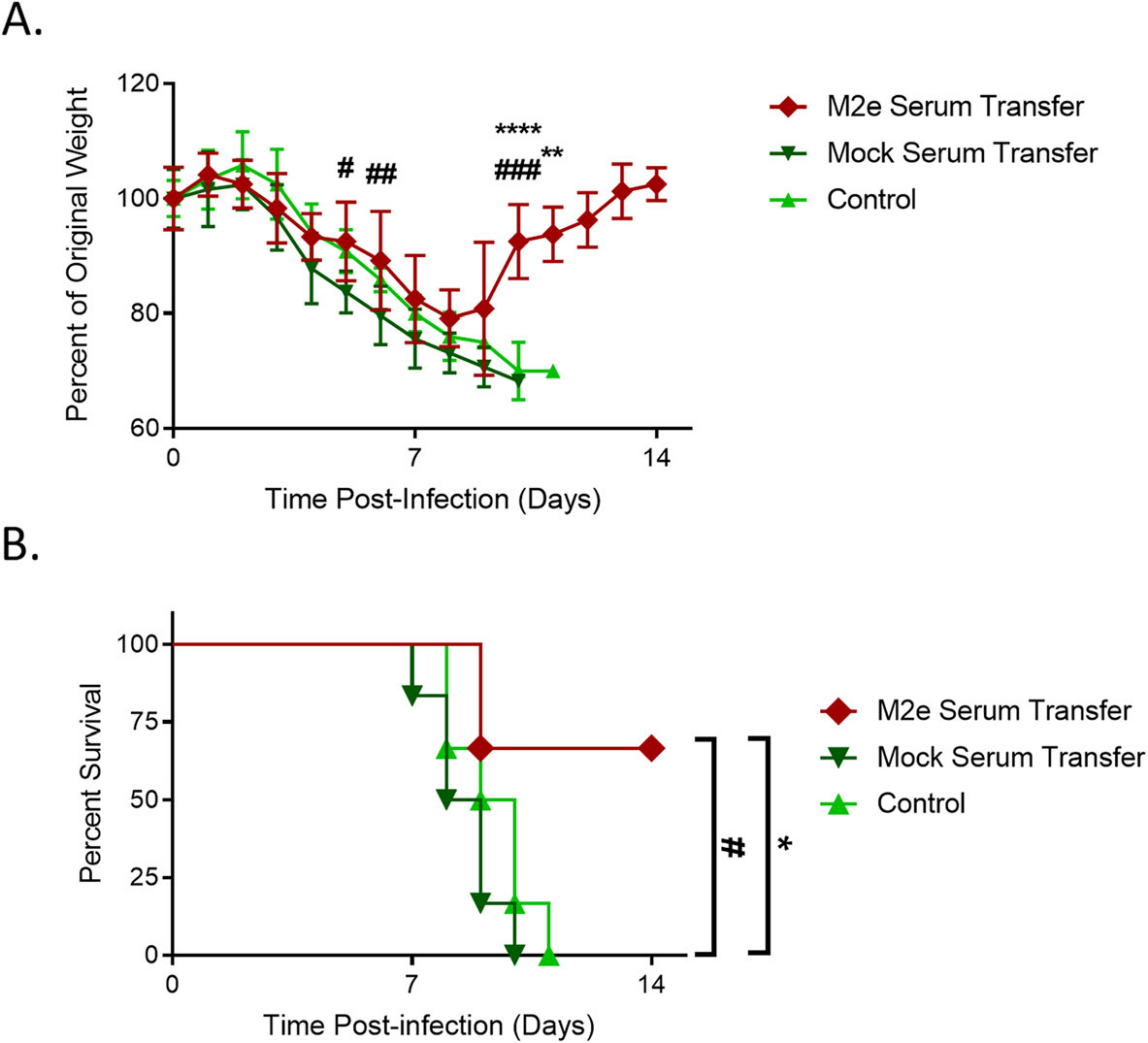
AuNP-M2e + sCpG vaccination induces antibody mediated protection. 6-8 week old BALB/c mice were immunized on Day 0 and Day 21 with 25 μL of AuNP-M2e + sCpG. On Day 42, serum was isolated from vaccinated mice via heart bleed and 300 μL of serum was transferred to naïve unvaccinated 6-8 week old BALB/c mice via intravenous injection. The passively immunized mice were challenged on Day 42 with 8.3 PFU A/PR/8/34 (H1N1). (**a**) Weight loss was monitored daily and percent weight loss was determined utilizing Day 0 Weight. *n* = 5–6, two-way ANOVA with a Dunnett’s multiple comparison test (comparisons directed towards M2e serum transfer group) and for day 11 only a Sidak’s multiple comparison test was used as mice from only 2 groups remained. (**b**) survival was monitored daily. n = 5–6, log-rank Mantel-Cox Test. * indicates significance compared to control group and # indicates significance compared to the mock serum transfer group. */# *p* < 0.05, **/## *p* < 0.01, ***/### *p* < 0.001, and ****/#### *p* < 0.0001

Considering the elevated levels of M2e-specific antibodies in protected vaccinated mice, we tested to see if serum M2e-specific antibodies could transfer protection to unvaccinated mice. BALB/c mice were vaccinated twice with AuNP-M2e + sCpG and their serum was isolated 21 days post-second vaccination at the time of peak serum antibody level. 300 μL of vaccinated or unvaccinated serum was transferred to naïve unvaccinated BALB/c mice prior to A/PR/8/34 (H1N1) infection (8.3 PFU). AuNP-M2e + sCpG vaccinated serum transfer significantly improved survival of passively immunized mice (Fig. 5). This data illustrates that the AuNP-M2e + sCpG vaccine does induce M2e-specific antibody-mediated protection. In combination with the maintained serum levels of M2e-specific antibodies in geriatric mice after aging and the elevation of these antibodies three months after infection as a result of activated memory B cells, this data suggests that M2e-specific antibodies are at least one mechanism of protection in geriatric mice.

## Discussion

Initial studies with AuNP-M2e + sCpG vaccination demonstrated protection against A/PR/8/34 (H1N1), A/California/04/2009 (H1N1pdm), A/Victoria/3/75 (H3N2), and A/Vietnam/1203/2004 (H5N1) [15, 20]. Additionally, the protection provided from AuNP-M2e + sCpG vaccination was demonstrated to persist 8 months post-vaccination in adult mice vaccinated as juveniles [21]. Our investigation establishes that AuNP-M2e + sCpG vaccination is protective after aging BALB/c mice to geriatric age.

To our knowledge, this is the first study examining serum antibody titers and establishing the efficacy of M2e-specific memory B cells after aging to geriatric age post-early vaccination. One study tested an M2 vaccine in an animal model after aging to geriatric age. This study by Garcia, et al. utilized an M2 DNA vaccine administered intranasally with recombinant adenoviruses (A/M2 rAd i.n.). This vaccine was shown to be about 50% protective in 20 month old mice, 17 months post vaccination [33]. However, antibody titers were never tested after aging more than one month post a vaccination boost and were only analyzed as total IgG. While we show a similar increase in protection between our vaccinated mice and our controls, we further analyze the serum of our mice for the subclasses of the M2e-specific antibodies, monitor them over time, and can see them elevate 3 months post infection indicative of functional memory B cells. Further, while other studies have examined longevity of protection and the isotype/IgG subclass makeup of M2e-specific serum, none have monitored those levels to geriatric age [21, 34, 35].

AuNP-M2e + sCpG vaccination produces a variety of M2e-specific antibody IgG subclasses which remain at elevated levels after aging. Before and after aging, we report significantly elevated levels of M2e-specific IgG1 and IgG2a, both of which induce immune responses critical for protection from influenza by activating a variety of Fc receptor mechanisms [34]. Additionally, despite a 15 month rest before infection, vaccinated mice maintained memory B cells which successfully elevated antibody titers in response to infection. At least in mice, it appears that immunosenescence does not inhibit the response by memory B cells induced by the AuNP-M2e + sCpG vaccination at a young age. Further, while titers decrease after aging, it does not appear that boosters would be necessary to maintain a protective memory response. Further supporting these conclusions, three times vaccinated mice 18 or 20 months old had no significant difference in M2e-specific antibody titers of any IgG subclass after the third vaccination, nor a significant difference in survival, despite the fact that in humans, this age difference would be a difference of 8 years representing the immune response of a ~ 65 year old and a ~ 73 year old (calculated based on the overall lifespan of mice and humans, based on post-senescence lifespan, this difference increases to represent nearly 38 human years) [31]. Rather the antibody titers were essentially the same, suggesting that the decreased antibody titer was more a direct result of the 15 month rest period, which represents over 50 years of aging in humans [31].

While the vaccine is protective against lethal influenza infection, it does not neutralize or prevent influenza infection in mice. Studies other models and perhaps in humans would have to further examine if and how this protection extends to improving influenza pathology. We do not see improved weight loss in geriatric mice, and while that is the traditional readout of disease severity in mice, it does not necessarily translate to breadth of symptoms humans experience as a result of influenza infection, nor even to the outcome of infection in those mice, as we see in our study. While weight loss often correlates to disease outcome in BALB/c mice, it does not in other strains [36]. These mice at the time of infection weighed an average of 24.0 g (Range: 19.0 to 26.4 g), which could affect their weight loss outcomes. Additionally, previous publications with the AuNP-M2e + sCpG vaccination in younger mice and with less time in between vaccination and challenge did show decreased weight loss in vaccinated controls [15, 20, 21]. Therefore, our data could be a result of the increased disease severity in geriatric aged mice or because of the length of time post-vaccination.

We further demonstrated that M2e-specific antibodies derived from AuNP-M2e + sCpG vaccination and transferred to naïve unvaccinated mice induce protection from lethal infection through passive immunization. These results support our hypothesis that M2e-specific antibodies derived from the AuNP-M2e + sCpG vaccine are at least partially responsible for the protection provided by this M2e vaccine and are consistent with the literature which consistently shows vaccine derived M2e-specific serum and monoclonal antibodies can transfer protection [12, 34, 35, 37].

Further studies will have to be done vaccinating naïve elderly mice with AuNP-M2e + sCpG to determine if AuNP-M2e + sCpG is an effective option for vaccinating adults over 65. Current vaccines are less effective in this age group, for example a study of the 2016–2017 influenza season in the United Kingdom found that while vaccination was 40% effective in adults between 18 and 65, the seasonal vaccine was not effective in adults over 65 [38]. Other studies have found seasonal or strain variance in the level of protection provided to people over 65 and have improved seasonal vaccine efficacy by increasing the dose of the vaccine, as well as, adding a number of adjuvants [26, 39]. Additionally, Garcia, et al. found that while A/M2 rAd i.n. could protect mice into geriatric age post-early vaccination, protection did not reach significance for mice vaccinated at 20 and 21 months of age and challenged at 22 months of age [33]. Only through further studying the vaccination of naïve elderly mice with AuNP-M2e + sCpG could the potential of this vaccine begin to be examined.

## Conclusions

M2e has long been considered an excellent target for a universal influenza A vaccine. The development of a universal vaccine has implications outside of our normal considerations for influenza with our seasonal vaccine, including the possibility of life long protection. To our knowledge this is one of the first studies testing this potential in an animal model. We utilized a published and potentially universal influenza A M2e vaccine, AuNP-M2e + sCpG, and vaccinated mice at an early age, monitored their M2e-specific immune response, and challenged them after the reached geriatric age. These mice maintained M2e-specific antibodies throughout aging. We found a variety of IgG subclasses present in the serum of geriatric aged mice after aging. Further, these amount of M2e-specific antibody present in the serum appeared to be related to two factors: first, the number of vaccinations given (e.g. mice receiving three vaccinations had more serum IgG at all time points, even after aging) and second, the time since vaccination (e.g. the amount of M2e-specific antibodies decreased as time past after vaccination). We did not find that increased age was directly responsible for any additional decrease in antibody titer apart from time post-vaccination, as mice vaccinated three times on the same day had equivalent antibody titers at all timepoints regardless of their being in an older or younger cohort (ages 20.8 and 18.2 months at time of challenge). Additionally, we found that M2e-specific antibodies from mice vaccinated with AuNP-M2e + sCpG were sufficient for protection. Together these data suggest that mice maintain protection against influenza A after aging despite decreasing antibody titer and that this protection is at least in part antibody mediated. These results reinforce the potential of M2e as an antigenic target for influenza A vaccination. If they translate to human studies, they suggest that if such an universal influenza A vaccine were established, it is possible that administration at an early age would provide life long protection from influenza A.

## Materials and methods

### Study design

**Fig. 6:**
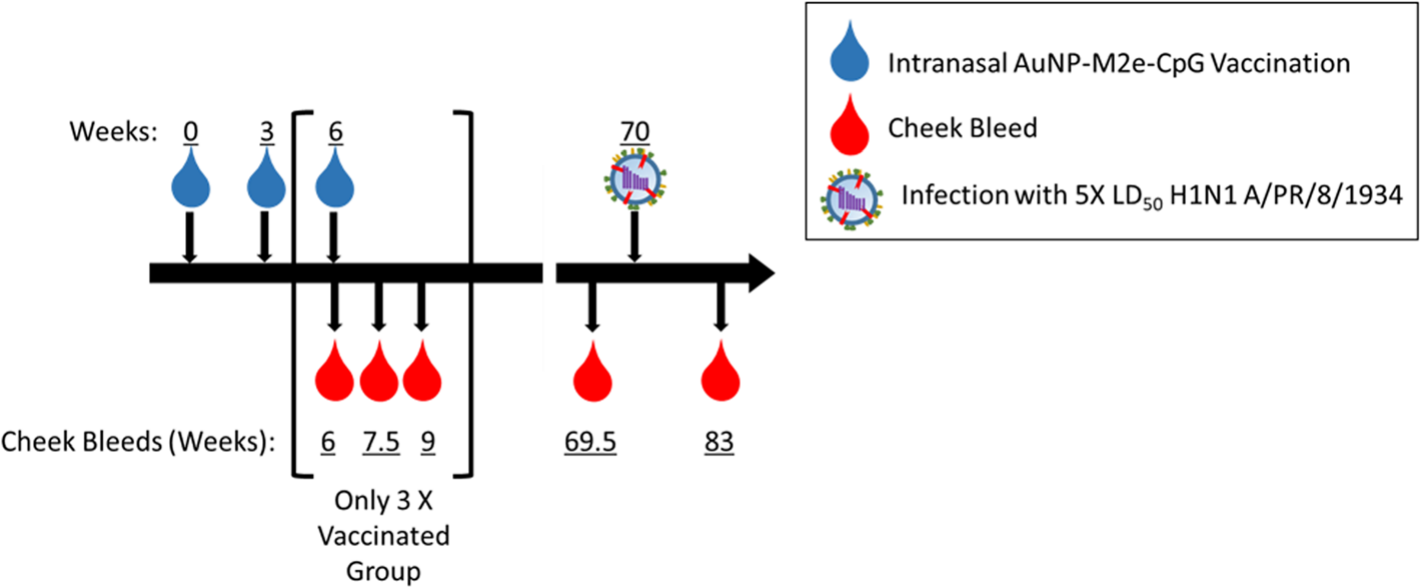
Experimental time points. A cohort of mice were vaccinated 0, 2, or 3 times with AuNP-M2e + sCpG vaccine and challenged after aging 15 months. Mice were bleed three times after the third vaccination (weeks 6, 7.5, and 9), prior to infection (week 69.5), and 3 months post infection (week 83)

For our purposes, we tracked the M2e-specific antibody titers in mice vaccinated two or three times between 3 weeks (0.8 months) and 19.6 weeks (4.9 months) of age with each vaccination 21 days apart. The mice were bled on the day of, 10 days after, and 21 days after the third vaccination, as well as, approximately 15 months after the third vaccination (3 days prior to infection) and 3 months after infection. Fig. 6 illustrates the overall course of the experiment and Table 1 indicates the ages of the mice within each group at each time point.

**Table 1:** Ages of mice at experimental time points

Group	# of Mice	Age (months)							
		AuNP-M2e-CpG 1	AuNP-M2e-CpG 2	AuNP-M2e-CpG 3	Bleed 2	Bleed 3	Bleed 4	H1N1 Infection	Bleed 5
				Bleed 1					
X3 (1)*	9	3.4	4.1	4.9	5.5	5.8	*20.6*	*20.8*	*23.8*
X3 (2)*	10	0.8	1.5	2.3	2.9	3.3	*18.1*	*18.2*	*21.3*
X2	19	0.8	1.5	–	–	–	*18.1*	*18.2*	*21.3*
Naïve	19	–	–	–	–	–	10.6	10.7	13.8
Experimental Time Point (weeks):		**0**	**3**	**6**	**7.5**	**9**	**69.5**	**70**	**83**

Mice were specified age in months at given time points (listed in weeks in bold). Mice over the age of 18 months are considered geriatric age (shown in *italics*). Groups that were not manipulated at a given time point have a dashed line ----. *3X Groups (1) and (2) were combined for all analysis as there was no significant difference between their antibody titer at any time point or their survival. (Data shown in Fig. 4g)

### Animals

BALB/c mice were bred internally from breeders obtained from Charles River Laboratories. Mouse ages are specified within information for given experiments. All mice were cared for in the animal facilities of the Center for Comparative Medicine at Baylor College of Medicine (BCM) and Texas Children’s Hospital (TCH), and all protocols were approved by the BCM Institutional Animal Care and Use Committee.

### AuNP-M2e + sCpG vaccination

The AuNP-M2e + sCpG, first described in Tao et al., 2014, is composed of 12 nm gold nanoparticles coated with a vaccine sequence M2e peptide vaccine through the gold-thiol interaction which conjugates the peptide to the gold nanoparticle, in this case binding the gold to an additional cystine on the C-terminal of the peptide. To avoid cross-linking of the nanoparticles through disulfide bonds, the vaccine sequence peptide was altered to substitute serine for the cystine at amino acid positions 17 and 19. These are the only 3 amino acid alterations distinguishing the AuNP-M2e + sCpG vaccine sequence (AA sequence: MSLLTEVETPIRNEWGSRSNDSSDC) from the consensus M2e sequence (AA sequence: MSLLTEVETPIRNEWGCRCNDSSD). The adjuvant sCpG is added to the vaccine and essential for the production of a robust antibody response to M2e [15].

AuNP-M2e + sCpG vaccine was prepared as described Tao et al., 2014 [15]. Mice were anesthetized with isoflurane and vaccinated dropwise intranasally with 25 μL of AuNP-M2e + sCpG vaccination (8.2 μg M2e, 60 μg AuNPs, and 20 μg sCpG per animal, as described in Tao et al., 2017) [20]. Mice were vaccinated two or three times, with each vaccination being 21 days apart. 3X vaccinated group included 19 mice (9 age 3.4 months at time of first vaccination, 10 age 3.5 weeks at time of first vaccination. Data was pooled). 2X vaccinated group included 19 mice (age 3.5 weeks at time of first vaccination).

### Determining antibody titer

Blood was collected from the submandibular vein on day of third AuNP-M2e + sCpG vaccination (week 6, 3X mice only), 10 days post third AuNP-M2e + sCpG vaccination (week 7.5, 3X mice only), 21 days post third AuNP-M2e + sCpG vaccination (week 9, 3X mice only), 15 months post third AuNP-M2e + sCpG vaccination date (week 69.5, all groups), and 3 months post H1N1 infection (week 83, surviving mice only).

Serum was isolated from the blood sample and frozen at − 30 °C. Serum samples from each mouse were individually analyzed in triplicate for antibody titer via ELISA (plate: Corning, Ref: 9018, Lot: 10017015). M2e vaccine peptide was used as the coating antigen for ELISAs (Vaccine sequence, specifications reported in Tao et al., 2014) [15]. Serum from specified mice and time point at a 1:3000 dilution was added, and M2e-specific titer was detected by an IgG subclass specific secondary antibody conjugated to HRP (Southern Biotech, Total IgG: 1030–05; IgG1: 1070–05; IgG2a: 1080–05; IgG2b: 1090–05; IgG3: 1100–05). The average background from naïve unvaccinated serum was subtracted as a control for each ELISA run. Absorbance was measured at 490 nm.

### Influenza a infection

Mice were anesthetized with isoflurane and vaccinated dropwise intranasally with 20 μL containing 8.3 PFU of A/PR/8/1934 (H1N1). 8.3 PFU is a 5XLD50 in 6-8 week old BALB/c mice. Mice were be weighed daily after serum transfer or infection.

### Virus

A/PR/8/1934 (H1N1) was obtained from ATCC and passaged through C57B6/J mice 10 times and BALB/c mice 6 times before isolation and then stored at − 80 °C.

### Serum transfer

Mice were vaccinated with AuNP-M2e + sCpG 2 times, 21 days apart (Days − 42 and − 21). On day 0, mice were euthanized using isoflurane and blood was collected via heart bleed. Serum was isolated from blood after the blood had coagulated. 300 μL of serum were transferred via tail vein intravenous (IV) injection into 10 naïve unvaccinated 6–8 week old mice. The recipient mice were subsequently infected with a 5XLD50 (8.3 PFU) of A/PR/8/1934 (H1N1) and monitored daily for survival and weight loss. For this experiment, mice were euthanized at 30% weight loss.

### Statistical analysis

All statistics were performed using Graphpad Prism 7. Possible triplicate outliers in ELISA results were tested via Grubbs’ Test with alpha = 0.2 and removed from data sets if confirmed as an outlier (approximately 2.6% of all sample triplicates contained a statistically verified outlier, representing < 1% of all replicates). For comparisons between of antibody titers between groups at a single time point, a one-way ANOVA with a Tukey’s multiple comparisons test was performed. Statistics for comparisons within groups comparing two time points utilized a paired T test. Finally, for comparisons two or more groups back to a single group, a two-way ANOVA with a Dunnett’s comparison was used. For comparisons between different numbers of samples, specified analysis was completed using row means with standard deviations calculated in Prism. Survival analysis utilized a Mantel-Cox log rank test. All statistics for a particular dataset are indicated in the figure legends. *p < 0.05, **p < 0.01, ***p < 0.001, and ****p < 0.0001.

## Data Availability

The datasets supporting the conclusions of this article are available in the Mendeley Data repository, 10.17632/7vhz76g4ng.1.

## Abbreviations

AuNP: Gold nanoparticles
CpG: Oligonucleotides that contain unmethylated CpG dinucleotides motifs (C:cytosine; G:guanine)
IgG: Immunoglobulin G
IgG1: Immunoglobulin G1
IgG2a: Immunoglobulin G2a
IgG2b: Immunoglobulin G2b
IgG3: Immunoglobulin G3
LD50: Lethal dose 50%
M2e: Extracellular domain of matrix protein 2
OD: Optical density
sCpG: soluble CpG
